# Clustering of chronic disease risk factors with tobacco smoking habits among adults in the work place in Sousse, Tunisia

**DOI:** 10.11604/pamj.2016.24.220.7163

**Published:** 2016-07-12

**Authors:** Hmad Sonia, Maatoug Jihene, Harrabi Imed, Ghammem Rim, Belkacem Mylene, Saadi Mounir, Amimi Souad, Knani Khaoula, Al’Absi Mustafa, Lando Harry, Mrizak Najib, Ghannem Hassen

**Affiliations:** 1 Department of Epidemiology, University Hospital Farhat Hached, Sousse, Tunisia; 2 Group of Occupational Medicine, Sousse, Tunisia; 3 University of Minnesota Medical School, USA; 4 Department of Epidemiology & Community Health, University of Minnesota, USA; 5 Department of Occupational Medicine, University Hospital Farhat Hached, Sousse, Tunisia

**Keywords:** Tobacco use, work places, risk factors, tobacco use, adults, chronic diseases

## Abstract

**Introduction:**

The aim of our study was to explore the major non-communicable risk factors (unhealthy diet, sedentarily, alcohol consumption) of smokers and nonsmokers in workplaces.

**Methods:**

A cross-sectional study was derived from an initial assessment in workplaces which was part of a community-based intervention to prevent chronic disease risk factors conducted in 2009 in the region of Sousse, Tunisia. The surveyed subjects were employees in six factories spread across three delegations in the region. Overall, 1770 of 2250 employees participated in the assessment. In this study, the clustering of non-communicable diseases risk factors with smoking habits was made only for male employees including in this study 1099 among 2250. Data were collected at worksites by a questionnaire, via interview or self-report. The main items assessed socio-demographics characteristics, smoking status, eating habits, level of physical activity and alcohol use of the participants.

**Results:**

The percentage of male smokers was 54.0%(n=594). Their average age of daily smoking initiation was 19.22 (±4.24 years). The percentage of male smokers consuming 5 fruits and vegetables per day was significantly lower than nonsmokers (57.2% vs 63.5%, p=0.04). The proportion of male smokers consuming alcohol was about three times that of nonsmokers (16.5% vs 5.8%, p=0.001). The proportion of male employees who agree with anti-smoking laws in work places was higher for nonsmokers than for smokers.

**Conclusion:**

A strong association existed between smoking and risky lifestyles factors in the work place. Such findings are potentially useful in directing intervention efforts regarding smoking cessation in occupational settings.

## Introduction

Tobacco use is a major health concern in the modern world [1, 2]. Based on recent estimates, nearly 20% of the world’s adult population smokes cigarettes. Tobacco use kills approximately 5.2 million people worldwide each year, a figure expected to increase to more than 8 million a year by 2030 [3, 4]. Likewise, current data indicate that Tunisia has one of the highest rates of adult male tobacco use in the Eastern Mediterranean region with a prevalence of 52.7% [5]. Furthermore, a growing occupational disparity in smoking prevalence represents a crucial challenge for worksite tobacco control initiatives [6].

Previous studies have reported that smoking is associated with various psychosocial and demographic factors including social status, education, age, employment and marital status [2, 7]. Some studies suggest that smoking acts both as a causal factor and as a marker of an unhealthy lifestyle [8, 9]. Moreover, the clustering of multiple risk factors provides support for multiple-behavior interventions as opposed to single-behavior interventions [10]. To date, the occurrence of smoking and other non communicable disease risk factors has not been fully investigated, particularly among workers in Tunisia. The clustering of lifestyle factors (primarily unhealthy diet, lack of physical activity, alcohol consumption) with smoking therefore needs further research. As such this study provides useful data concerning smoking patterns among adults in the work place in the region.

## Methods

**Study design:** This is a cross-sectional study which represents a part of an assessment of the prevalence of chronic disease risk factors in workplaces. This assessment was part of a community-based intervention to prevent chronic disease risk factors carried out in 2009 in the region of Sousse, Tunisia.

**Population:** The pre-assessment of the community-based intervention took place in the manufacturing sector in six factories spread across three delegations of the region of Sousse. All employees in these six factories were included. Overall, 1770 from 2250 employees participated at the initial assessment. However, as we already know that women seemed often to underreport their smoking status due to social issues, which might distort the clustering of risk factors with smoking habits, we have chosen in the current study, to focus only on male employees. The number of male employees among the 2250 was 1099. They were all included in this study.

**Data collection:** Socio-demographic characteristics and lifestyle data have been collected by a pre-tested questionnaire in Arabic: the managerial staff responded to a self administrated questionnaire, while workers were interviewed by a medical doctor. Socio-demographic characteristics measured included age, sex, marital status, educational level and profession. Lifestyle items were composed of smoking status, daily cigarette consumption, eating habits, physical activity and alcohol consumption. Concerning smoking habits, the participants were asked if they smoked any kind of tobacco (cigarettes, cigar, pipe or water-pipe). “Smokers” were the participants who responded yes to this question. BMI was determined as the body weight in kilograms divided by squared height in meters (kg/m^2^). Obesity was defined as a BMI ≥30 kg/m^2^. Analysis: Statistical analysis was performed using the SPSS 10.0 software. Data were presented as frequencies, means and standard deviations. The chi-square test was used to compare current smokers and never smokers for the categorical variables. The t-test was used to compare the means and standard deviation (SD) of quantitative data. For both tests, the level of significance was 0.05.

## Results

The mean age of male employees was 33.8±8.59 years. The percentage of male smokers was 54.0 % (n=594). The average age of initiation of daily smoking was 19.22 (±4.24 years). Their mean daily consumption of cigarettes was 16.4±9.37. As indicated in Table 1, a higher percentage of male employees with a primary level of education reported smoking than did employees with university level of education (63.3% vs 49.1%, p =0.007). The proportion of smokers was also significantly higher among single employees than among married employees (61.1% vs 53.4%, p=0.002). The proportion of smokers among male workers (58 %) was higher than that among male managerial staff (46.6%), but this difference was not statistically significant (p=0.39).

**Table 1:** Socio-demographic characteristics of male workers according to their smoking status

	Non-smokers, n (%)	Smokers, n (%)
***Education level***		
Primary	68(36.7)	117(63.3)
Secondary	301(44.5)	376(55.5)
University	88(50.9)	85(49.1)
***Profession***		
Managerial staff	62(53.4)	54(46.6)
Technician	60(48.0)	65(52.0)
Workers	320(41.7)	447(58.3)
***Marital status***		
Married	319(46.6)	365(53.4)
Single	143(39.9)	215(61.1)
Divorced	2(25)	6(75)

Male smokers and nonsmokers differed significantly in lifestyle habits (Table 2). The percentage of male smokers consuming 5 fruits and vegetables per day was significantly lower than male nonsmokers (57.2% vs 63.5%, p=0.04). The percentage of male smokers avoiding foods that are too fat or too sweet or too salty was also significantly lower than nonsmokers male (41.9% vs 53.9%, p=0.001). The percentage of smokers adding salt to meals was significantly higher than nonsmokers with 29.3% vs 21.2%, p=0.003. Moreover, the percentage of male smokers consuming alcohol was about three times the percentage of non smokers male consumers (16.5% vs 5.8%, p=0.001). Concerning physical activity, we found a significant difference between male smokers and male nonsmokers, who tried to reduce time setting outside work (25.7% vs 33.7%, p=0.005). Only 31.0% of male smokers and 36.4% of male nonsmokers (p=0.06) used to practice physical activity 30 minutes or more per day, 5 days per week. However, as might be expected, the rate of obesity (defined as BMI≥30kg/m^2^) was significantly higher among male nonsmokers than male smokers (19.4% vs 14.2%, p=0.02)

**Table 2:** Lifestyles habits of male workers according to their smoking status

	Non-smokers, n (%)	Smokers, n (%)	p: degree of significance
Consuming 5 fruits and vegetables per day	290(63.5)	332(57.2)	0.04
Adding salt to meals	98(21.2)	172(29.3)	0.003
Avoiding foods that are too fat or too sweet or too salty	251(53.9)	244(41.9)	0.001
I consume alcohol	26(5.8)	94(16.5)	0.001
I practice physical activity 30 minutes or more per day, 5 days per week	168(36.4)	182(31.0)	0.06
I try to reduce sedentary activity outside work	155(33.7)	151(25.7)	0.005

Table 3 presents the proportion of male employees who agree with anti-smoking laws in work places. Although nonsmokers were more likely to agree, there was substantial agreement among smokers as well (respectively 87.6% vs 73.0%, p=0.001). However, the proportion of male workers who reported that their company encourages them to prevent or stop smoking was similar for both smokers and nonsmokers (25.1% vs 23.2%, p=0.47).

**Table 3:** Male workers’ attitudes concerning smoking in work places according to their smoking status

	Non-smokers, n (%)	Smokers, n (%)	p: degree of significance
I agree with anti-smoking law in the workplace	403(87.6)	424(73.0)	0.001
your company encourages you to prevent or stop smoking	104(23.2)	146(25.1)	0.47

## Discussion

Aiming for more investigation on the clustering of risk factors with smoking habits, we have analyzed data from a cross sectional survey conducted in the work place. The results highlighted the important magnitude of tobacco use in work places and demonstrated a strong association between smoking and risky lifestyles, such as unhealthy diet, alcohol consumption and lack of physical activity.

The reported prevalence of smoking was 54% among male employees and this is relatively consistent with previous Tunisian statistics. A study carried out in the region of Ariana found prevalence smoking of 55% in men [11]. Another study conducted in the Tunisian Sahel reported a percentage of 66% of men who are smokers [12]. Worldwide, the prevalence of smoking is much higher in men than in women [13, 14] and the pattern is similar in the Mediterranean region where the proportion of men who smoke ranges from 20% in Islamic Republic of Iran to 63% in Turkey [15].

The average age of initiating daily smoking found among male employees (19.2 years) was similar to another Tunisian survey [15]. Though, Lando et al [16] have found 17.7 years the mean age of initiation in a study involving 2120 working adults, they noted that those who initiate smoking at an early age may be at higher risk for poor dietary practices, reduced physical activity, greater alcohol consumption, and a variety of other risky health practices [16]. Our results revealed mean daily consumption of 16.4±9.37 cigarettes among male workers and this is quite similar to Lando’s et al results with 16.7±9.2 [16]. Smoking and educational level seem to be strongly associated [2]. Many studies have demonstrated that smoking is more prevalent in less educated people [2, 15, 17]. However, significant international variations in educational gradients and smoking may exist. Similar findings were documented in our study. In fact, primary level education was more frequent among smoking employees than among nonsmokers. Employees with university level education were less likely to smoke. Thus, the prevalence of smoking and educational level were inversely related.

Smoking prevalence depends on social class and occupation [18]. In western countries, tobacco use is more frequent in the lower socioeconomic classes [2]. In Tunisia, Fakhfakh et al [15], have reported this fact in an epidemiological study including 1334 adults where smoking among manual workers were 54.4%. As to our results, we have found that lower grade male employees (workers) were likely to be current smokers (58.3%) but not significantly different from higher grade male employees (managerial staff) being smokers (46.6%). Similarly a Japanese study [2] has obtained similar findings and attested that lower income generally promotes smoking with the strongest influence in the 25-39 years population group.

Regarding lifestyle habits, our results indicated that several variables were associated with smoking status. Indeed, male smoking employees appeared to have a less healthy diet, as the percentage of smokers consuming 5 fruits and vegetables per day was significantly lower than nonsmokers in our studied population. This has been widely demonstrated in the literature. Smokers compared with never-smokers have been shown to eat less cereal [19], less vegetables and fruits, and eat more fat [19–21]. Furthermore, smokers have been shown to have a higher energy intake [8]. Philips et al [21] have demonstrated from a cross-sectional household health survey that smoking status was inversely associated with intake of vitamins A and C, dietary fiber, folate, and iron among women, whereas differences were smaller and not significant among men. Women who smoked consumed fewer servings of fruits and vegetables than nonsmokers, but this trend was not noted in men. In a Japanese study involving 2310 workers, comparing the lifestyle of smokers with non-smokers, smokers had worse health practices than nonsmokers with regard to eating breakfast and nutritional balance [22].

On the other hand, in most populations, smokers weigh less than do nonsmokers [23, 24]. Adult smokers weigh, on average, 4-5 kg less than nonsmokers and are less likely to be overweight or obese [25]. In Finnish men, the relation between smoking and BMI was reported to change from an inverse association to a positive one in the late 1980s [24]. Results have demonstrated that smoking employees are significantly less obese compared with non-smoking employees.

However, according to Thompson et al [23], weight differences between smokers and nonsmokers are more likely to be due to differences in diet than in exercise.

Certainly, smoking and physical activity are largely incongruent behaviors. Considerable interest currently exists in how physical activity indirectly influences health by acting through other behaviors, such as smoking [26]. The association between cigarette smoking and poor physical functioning has been demonstrated in the published literature [2]. Woodward et al. [27] reported that smokers had lower physical activity than non- smokers [22]. Hu et al [2] have demonstrated that civil servants who did not engage in regular moderate physical activities (more than twice per week) are 1.4 times more likely to be active smokers. In a similar Italian study among civil servants, the percentage of sedentary subjects was found to be particularly high among smokers (67%) versus nonsmokers (59%) [28]. Our results show that the percentages of smokers who practice recommended levels of physical activity (30 minutes or more per day, 5 days per week) were non-significantly lower than the percentages of nonsmokers who met this standard. Whereas, the percentages of smokers and non-smokers, who tried to reduce time setting outside work were significantly different. These findings appear relatively controversial to other studies. In fact, in a review of the literature, Blair et al, noted that smoking may be inversely associated with leisure-time activity and that occupational physical activity is positively associated with the smoking habit, although this is likely due to confounding by socioeconomic status [26]. However, they documented several problems encountered in reviewing research on exercise and physical activity. First, there are no consistent definitions of these terms used by different authors. Also, assessments of exercise and physical activity have been crude and imprecise; therefore, much misclassification occurs in studies of exercise behavior and, in turn, this may obscure the relationships between exercise or physical fitness and other behaviors [26].

Strong association between smoking behavior and alcohol consumption was demonstrated in this study. A strong positive relationship between alcohol and tobacco smoking previously has been reported [2, 29, 30]. A cross-sectional study carried out among 4521 male Japanese workers demonstrated that subjects who consumed the lowest amount of alcohol had the lower prevalence of smoking [29]. An epidemiological analysis of alcohol consumption in the USA [30] revealed that among young adults, the prevalence of recent concurrent alcohol and tobacco use was approximately 35 to 45 percent.

Other interesting results of this study are those that have implications for smoking intervention programs. In fact, the primary objective of workplace smoking restrictions and bans may be to reduce environmental tobacco smoke in the workplace. However, several studies have shown that restrictions and bans also lead to a decrease in the prevalence of smoking and the number of cigarettes smoked daily among workers [31]. Workers’ attitudes regarding the implementation of an anti-smoking law at worksites therefore need to be addressed. Research suggests that both nonsmokers and smokers at work prefer a smoking policy to be in place [32]. Our results were in accord with this as the majority of non-smoking and smoking workers support anti-smoking policies. Pearson D et al [33] have reported in a study involving hospitality workers that more than half of all workers supported local bans on smoking in restaurants, in bars, and on a statewide basis. They preferred to work in smoke-free settings and a majority supported bans on smoking in the workplace [33]. On the other hand, providing prevention programs for tobacco cessation in workplaces would be more effective. Sherriff NS et al [34] reported that employees were knowledgeable about the negative health impacts of smoking, but showed limited awareness of smoking cessation services and aids available. Our findings are in accord as the proportions of both smokers and nonsmokers who were knowledgeable about their companies’ encouragement of prevention and smoking cessation were quite limited.

It’s important to address the limitations of this study, which are mainly associated with the design factors. Indeed, it may be difficult to generalize the findings to different settings and populations as the studied population wasn’t randomly selected. A second limitation is self-reported smoking status (without objective measures). The validity of self-reporting in determining the rate of smoking remains often questioned. Moreover, we haven’t assessed the clustering of risk factors among women as our study may have underestimated the rate of female smoking, (there may have been under-reporting in spite of assurances to participants about the confidentiality of the data).

Finally, health monitoring at the workplaces could contribute to a better awareness of these risky lifestyle factors, independently of workers’ socioeconomic level. Regular data collection on tobacco use should be thenceforth disaggregated by sex. This will permit identification of trends and health effects on men and women and also will illuminate the impact of gender on smoking initiation, types of tobacco used, and depth and frequency of inhalation. The research tools of estimating smoking prevalence, such as self-reporting, must be improved, as women may be more reluctant to report accurately due to greater social disapproval of female smoking in our society [35].

## Conclusion

Our study suggests the importance of considering differences in lifestyle risk factors in relation to smoking status to improve lifestyle practices among workers. Further investigation should be conducted in order to focus on an effective strategy for smoking cessation in these groups. These are very important issues for occupational health in Tunisia.

### What is known about this topic

- Tunisia has one of the highest rates of adult tobacco use in the Mediterranean region;
- The disparity in smoking prevalence in workplaces;
- Workplaces could be an interesting setting to assess and intervene on unhealthy life styles.

### What this study adds

- The important prevalence of tobacco use at workplaces mainly among men;
- The strong association between smoking and risky life styles factors;
- The importance of preventing all risk factors in association with tobacco use to be more effective.
